# Ultrahigh Energy Storage Density in Glassy Ferroelectric Thin Films under Low Electric Field

**DOI:** 10.1002/advs.202203926

**Published:** 2022-09-18

**Authors:** Yunlong Sun, Le Zhang, Qianwei Huang, Zibin Chen, Dong Wang, Mohammad Moein Seyfouri, Shery L. Y. Chang, Yu Wang, Qi Zhang, Xiaozhou Liao, Sean Li, Shujun Zhang, Danyang Wang

**Affiliations:** ^1^ School of Materials Science and Engineering The University of New South Wales Sydney NSW 2052 Australia; ^2^ School of Power and Energy Northwestern Polytechnical University Xi'an 710129 China; ^3^ School of Aerospace, Mechanical and Mechatronic Engineering The University of Sydney Sydney NSW 2006 Australia; ^4^ Department of Industrial and Systems Engineering The Hong Kong Polytechnic University Hong Kong China; ^5^ Frontier Institute of Science and Technology and State Key Laboratory for Mechanical Behavior of Materials Xi'an Jiaotong University Xi'an 710049 China; ^6^ Electron Microscope Unit Mark Wainwright Analytical Centre The University of New South Wales Sydney NSW 2052 Australia; ^7^ Solid State & Elemental Analysis Unit Mark Wainwright Analytical Centre The University of New South Wales Sydney NSW 2052 Australia; ^8^ Institute for Superconducting and Electronic Materials AIIM University of Wollongong Wollongong NSW 2500 Australia

**Keywords:** energy storage, glassy ferroelectrics, lead‐free thin films, morphotropic phase boundary, super tetragonal nanostructures

## Abstract

The current approach to achieving superior energy storage density in dielectrics is to increase their breakdown strength, which often incurs heat generation and unexpected insulation failures, greatly deteriorating the stability and lifetime of devices. Here, a strategy is proposed for enhancing recoverable energy storage density (*W*_r_) while maintaining a high energy storage efficiency (*η)* in glassy ferroelectrics by creating super tetragonal (super‐T) nanostructures around morphotropic phase boundary (MPB) rather than exploiting the intensely strong electric fields. Accordingly, a giant *W*_r_ of ≈86 J cm^−3^ concomitant with a high *η* of ≈81% is acquired under a moderate electric field (1.7 MV cm^−1^) in thin films having MPB composition, namely, 0.94(Bi, Na)TiO_3_‐0.06BaTiO_3_ (BNBT), where the local super‐T polar clusters (tetragonality ≈1.25) are stabilized by interphase strain. To the knowledge of the authors, the *W*_r_ of the engineered BNBT thin films represents a new record among all the oxide perovskites under a similar strength of electric field to date. The phase field simulation results ascertain that the improved *W*_r_ is attributed to the local strain heterogeneity and the large spontaneous polarization primarily is originated from the super‐T polar clusters. The findings in this work present a genuine opportunity to develop ultrahigh‐energy‐density thin‐film capacitors for low‐electric‐field‐driven nano/microelectronics.

## Graphical abstract

A strategy is proposed for enhancing recoverable energy storage density (*W*_r_) while maintaining a high energy storage efficiency (*η)* in glassy ferroelectrics by creating super tetragonal (super‐T) nanostructures around morphotropic phase boundary rather than exploiting the intensely strong electric fields.

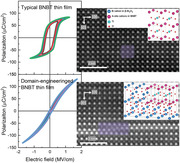

## 1 Introduction

Dielectric capacitors with ultrahigh power densities are highly sought‐after fundamental energy storage components in electronic devices, mobile platforms, and electrical pulsed power systems.^[^ ^1^, ^2^ ^]^ Electrostatic capacitors based on dielectric thin films are of particular interest for use in microelectronic circuits and miniaturized power devices. Despite their ultrafast charge–discharge rate as well as superior thermal and mechanical stability in contrast to electrochemical capacitors and batteries, the energy storage density of dielectric capacitors is generally low.^[^ ^3^ ^]^ The increased functionality of modern devices requires higher energy density and better charge/discharge efficiency of dielectric capacitors than the state of the art. Therefore, extensive efforts have been devoted to improving their energy densities fulfilling the demands of advanced electronic devices and electrical systems for integration, compactness, and miniaturization.

According to the relation $W_{r}=\int_{P_{r}}^{P_{m}}E\;dP$, the recoverable energy storage density *W*_r_ is determined by the maximum polarization (*P*_m_), remnant polarization (*P*_r_), and the driving electric field *E*.^[^ ^4^ ^]^ The existing attempts to improve the energy density of ceramic films are primarily centered on enhancing their dielectric breakdown strength *E*_b_, given *W*_r_ increases almost linearly with the increase of *E*_b_.^[^ ^5^ ^]^ Various approaches have been adopted to increase the *E*_b_ of dielectric thin films, such as interfacial modification, introduction of seeding/capping layer, design of polymorphic nanodomain, superparaelectric phase, etc.^[^ ^6^, ^7^, ^8^, ^9^, ^10^ ^]^ These approaches unambiguously led to impressive energy storage performance. For instance, an ultrahigh *W*_r_ of 152 J cm^−3^ with an efficiency >80% has been obtained under 5 MV cm^−1^ in Sm‐doped 0.3BiFeO_3_‐0.7BaTiO_3_ thin films featuring superparaelectric relaxor behaviors.^[^ ^10^ ^]^ Nonetheless, the requirement of the substantially high electric field (3–5 MV cm^−1^) to obtain high energy densities raises serious concerns over corona leakage, Joule heating, electrical fatigue, and catastrophic dielectric breakdown, in particular at elevated temperatures, and also brings challenges for the supporting insulation system, which may limit their applications on miniaturized equipment with the high level of integration.^[^ ^11^ ^]^ It is obvious that high energy densities do not necessarily translate into practical energy‐storing devices owing to the aforementioned high‐field‐induced issues. Practically it is of great importance to reduce the applied electric field to the energy‐storing working body. More importantly, the rapid development of implantable and wearable electronics, such as heart defibrillators and implantable hearing AIDS, triggers a greatly increased demand for energy‐storing devices with comparable energy density at finite electric field strength.^[^ ^12^, ^13^ ^]^ Thus, an alternative strategy for achieving giant energy density, rather than exposing the dielectric thin films to an excessive electric field, is highly desired.

**Figure 1:**
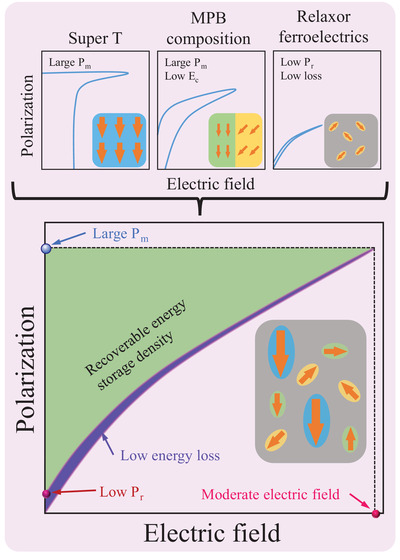
Schematic diagram showing the design rationale of high energy density *W*_r_ (green part), low loss (purple part) under a moderate electric field in dielectric thin films by coupling glassy ferroelectrics having MPB composition and super‐T nanostructures, where yellow, green, and blue ellipses represent rhombohedral, tetragonal, and super tetragonal polar clusters, respectively.

Excellent energy storage performance can also be yielded under a moderate electric field through boosting up (*P*_m_ − *P*_r_) in a judiciously designed relaxor thin film. The presence of relaxors features (also known as glassy ferroelectrics) can drastically decrease the *P*_r_ and hysteresis loss, potentially resulting in an increase in *W*_r_ and energy efficiency *η* ( = $W_{r}/W$, $W\;=\int_{0}^{P_{m}}E\;dP$).^[^ ^14^ ^]^ Efforts have been made to improve *η* through reducing the hysteresis. For instance, Silva et al. obtained a highly linear hysteresis loop with low energy dissipation in 0.5Ba(Zr_0.2_Ti_0.8_)O_3_–0.5(Ba_0.7_Ca_0.3_)TiO_3_ thin films through introducing an internal depolarization field via the insertion of a thin HfO_2_:Al_2_O_3_ dielectric layer.^[^ ^15^ ^]^ Nevertheless, further enhancement in *W*_r_ of glassy ferroelectrics heavily relies on the ultimate improvement of *P*_m_. Zhang et al. confirmed that the construction of a relaxor‐morphotropic phase boundary (MPB) crossover favors a modest growth in *P*_m_ without the need for a large electric field.^[^ ^16^ ^]^ In spite of that, a more effective way to upraise *P*_m_ of ferroelectric thin films is to introduce a super tetragonal (super‐T) phase with very large *c/a* ratios.^[^ ^17^, ^18^, ^19^ ^]^ Epitaxial strain arising from the lattice mismatch between the substrate and thin film materials is a well‐established means to produce super‐T structures. For example, sizeable *c*/*a* ratios of ≈1.1 and ≈1.2 were obtained in PbZr_0.2_Ti_0.8_O_3_ thin film^[^ ^20^ ^]^ and BiFeO_3_ thin film^[^ ^21^ ^]^ deposited on (001) SrTiO_3_ and (001) YAlO_3_ single crystal substrates, respectively. Nevertheless, a high level of lattice misfit strain imposed by the substrate can only be sustained within a critical thickness, i.e., several tens of nanometers, beyond which strain relaxation occurs, leading to the collapse of super‐T structures. Another viable route for creating super‐T structures is to utilize interphase strain in nanocomposite‐like thin films. Such super‐T structures are induced by mismatch in lattice parameter *c* between the thin‐film material and a secondary phase precipitated due to a local chemical inhomogeneity, such as PbO in PbTiO_3_ thin films,^[^ ^17^ ^]^ *β*‐Bi_2_O_3_ in BiFeO_3_ thin films,^[^ ^22^ ^]^ and BaO in BaTiO_3_ thin films.^[^ ^23^ ^]^ It should be noted that the interphase strain can be preserved in a film that is much thicker than the critical thickness if the processing conditions are judiciously controlled. Therefore, the approach of creating super‐T nanopolar clusters by utilizing interphase strain can be considered a universal tool to modify the functional properties of oxide perovskite thin films. It is feasible to a broad range of thin films, not only those with volatile elements such as Bi‐ and Pb‐based thin films but also families merely possessing nonvolatile elements such as BaTiO_3_ thin films. Given the facts outlined above, the introduction of super‐T nanostructures into glassy ferroelectrics with MPB composition would be a feasible solution to produce a giant energy storage density under a low‐to‐moderate electric field, as shown in **Figure** **1**.

In this work, an exceptional room‐temperature energy storage performance with *W*_r_ ∼ 86 J cm^−3^, *η* ∼ 81% is obtained under a moderate electric field of 1.7 MV cm^−1^ in 0.94(Bi, Na)TiO_3_‐0.06BaTiO_3_ (BNBT) thin films composed of super‐T polar clusters embedded into normal R and T nanodomains. The super‐T nanoclusters with a *c*/*a* ratio up to ≈1.25 are stabilized through interphase strain offered by the secondary phase of *β*‐Bi_2_O_3_ at an appropriate concentration, allowing a sharp escalation in (*P*_m_ − *P*_r_).

## 2 Results and Discussion

**Figure 2:**
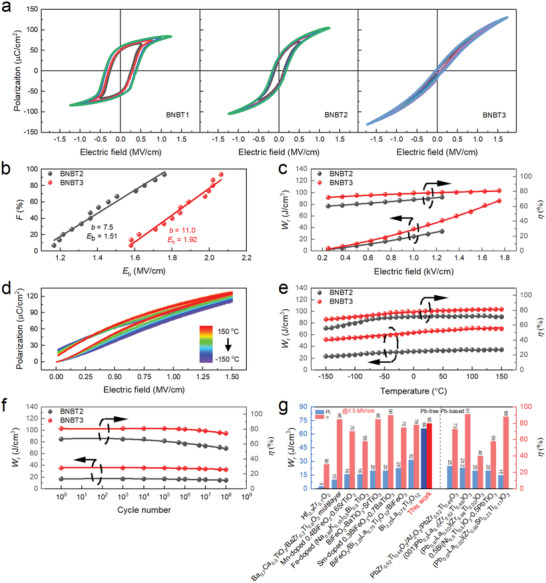
a) The *P–E* hysteresis loops of BNB1, BNBT2, and BNBT3 thin films under various electric fields at room temperature. b) Two‐parameter Weibull distribution analysis of the breakdown strengths of BNBT2 and BNBT3 thin films. c) The electric field‐dependent recoverable energy storage density and efficiency of BNBT2 and BNBT3 thin films. d) Temperature‐dependent unipolar *P*–*E* loops of BNBT3 under 1.5 MV cm^−1^. e) Thermal stability of recoverable energy density and efficiency of BNBT2 and BNBT3 under an *E* = 1.5 MV cm^−1^ over a temperature range of −150 to 150 °C. f) Room‐temperature fatigue performance of BNBT2 and BNBT3 under *E* = 1 MV cm^−1^ with regard to the 50 kHz charging and discharging cycles. g) Comparison of energy storage density and corresponding efficiency of typical Pb‐free and Pb‐based dielectric thin films under *E* = 1.5 MV cm^−1^.

Three thin‐film samples referenced as BNBT1, BNBT2, and BNBT3, respectively, of the same nominal composition, were prepared on SrTiO_3_ (001) single‐crystal substrates by laser molecular beam epitaxy (LMBE). Details of the processing parameters of each sample were given in the Experimental Section. The room‐temperature polarization–electric field (*P*–*E*) hysteresis loops under various electric fields are shown in **Figure** **2**a. BNBT1 resembles a typical ferroelectric behavior as evidenced by its rather square *P*–*E* loops consistent with its MPB composition. In stark contrast, the fast decaying polarization near the zero‐field on the *P*–*E* loops of BNBT2 and BNBT3 indicates the increase of the ergodic relaxor phase, coinciding with the steadily increased relaxor diffuseness factor *γ* as displayed in Figure S1 in the Supporting Information, where the greatest value of 1.88 is observed for BNBT3. On that account, the remnant polarization *P*_r_ dramatically drops from 59 µC cm^−2^ in BNBT1 to 7.0 µC cm^−2^ in BNBT3, which is conducive to a higher energy storage efficiency. On another note, these two samples exhibit a considerably increased *P*_m_, in particular, the *P*_m_ of BNBT3 is 131 µC cm^−2^ compared with BNBT1. The reduction in *E*_c_ of BNBT2 and BNBT3 suggests the decrease in the required energy barrier for domain switching, which is also well documented by the piezoresponse force microscopy (PFM) results (Figure S2a–c, Supporting Information), where the voltage required for the local reversal of the domains reduces significantly from 3.5 V for BNBT1 to 1.5 V for BNBT3. The rapid back‐switching of domains upon removing the poling electric field as shown in the “box‐in‐box” DART‐PFM images (Figure S2e,f, Supporting Information) further underpins the formation of relaxor phases in BNBT2 and BNBT3.

Among the two samples exhibiting slim *P*–*E* loops, the statistical breakdown strength *E*_b_ of BNBT3 is marginally higher than that of BNBT2 as shown in Figure 2b according to the two‐parameter Weibull distribution analysis based on $F\;=\;1-\exp(-{\left(E_{i}/E_{b}\right)}^{\beta })$, where *β* is the Weibull parameter, *E*_i_ is the measured breakdown strength, and *F* is the probability of electric breakdown at *E*_i_. Figure 2c gives the evolution of *W*_r_ and *η* of BNBT2 and BNBT3 thin films as a function of the applied electric field. *W*_r_ increases almost linearly with *E* for both samples. Remarkably, *W*_r_ and *η* of BNBT3 are up to ≈86 J cm^−3^, ≈81% under a moderate *E* = 1.7 MV cm^−1^. In addition, *W*_r_ and *η* of BNBT3, which are derived from the temperature‐dependent unipolar *P*–*E* loops under *E* = 1.5 MV cm^−1^ (Figure 2d), display good thermal stability over a wide temperature span, as shown in Figure 2e. From −50 to 150 °C, which is within the temperature window of technical importance of most electronic devices, a variation in *W*_r_ and *η* of BNBT3 was found to be ± 7% and ± 4%, respectively, compared with the respective value at room temperature. In particular, the BNBT3 sample can retain an extremely high *W*_r_ of >70 J cm^−3^ and *η* of ≈81% at 150 °C, confirming the capability of the studied thin film to work as high‐energy‐density energy‐storing capacitors under harsh conditions. The temperature insensitivity of energy storage properties is attributed to the enhanced degree of relaxor behavior of BNBT3, leading to a diffuse phase transition (Figure S3, Supporting Information) with much mild temperature dependence of the ferroelectric properties. Moreover, the relatively low leakage current density (<10^−3^ A cm^−2^, Figure S4, Supporting Information) suppresses the thermal activation of carriers and diminishes the dielectric loss, thus contributing to a remarkable energy storage efficiency. The charging and discharging cycle measurement is conducted under an AC electric field of *E* = 1.0 MV cm^−1^ (Figure 2f). To accomplish the fatigue test in an accelerated timeframe (<6 h) to mitigate the impacts of extrinsic factors during measurement, a charging and discharging frequency of 20 kHz is adopted.^[^ ^24^ ^]^ After 10^8^ cycles at room temperature, the energy storage density and efficiency of BNBT3 show a minor degradation of <8%, demonstrating excellent fatigue endurance.

The room‐temperature energy storage performance of a number of typical Pb‐free and Pb‐based thin films under a finite electric field (1.5 MV cm^−1^) is summarized in Figure 2 g. A low‐to‐moderate electric field is often preferred for dielectric energy storage, reducing the probabilities of breakdown and benefiting the operation reliability and cycling lifetime of the device. It is clear that the studied BNBT3 thin film far outperforms all the reported dielectric thin films in terms of energy storage density under such a moderate electric field. To further elucidate the competitive advantage of our strategy in lifting energy storage properties under a moderate electric field, the recoverable energy storage density and efficiency, corresponding strength of the applied electric field, and (*P*_m_ – *P*_r_) value of some best performing dielectric thin films are listed in **Table** **1**. It is evident that the superb energy storage performance of the studied BNBT3 thin film largely originated from their huge (*P*_m_ – *P*_r_) value of 124 µC cm^−2^ under a moderate electric field of 1.7 MV cm^−1^, substantially larger than those of state‐of‐the‐art thin films driven by desperately high electric field up to ≈5 MV cm^−1^.

**Table 1:** Comparison of energy storage performance and related (*P*_m_ – *P*_r_) value of best performing dielectric thin films to date. Note: FE: ferroelectric, AFE: antiferroelectric, RFE: relaxor ferroelectric, SPE: superparaelectric

Thin films	*W*_r_ [J cm^−3^]	*η* [%]	*E*_max_ [MV cm^−1^]	*P*_m_ [µC cm^−2^]	*P*_r_ [µC cm^−2^]	*P*_m_ – *P*_r_ [µC cm^−2^]	Ref.
(100) Ba(Zr_0.2_Ti_0.8_)O_3_ (FE)	166	80.0	5.7	60	10	50	[25]
Pb_0.5_Sr_0.5_HfO_3_ (AFE‐PE)	77	97.0	5.1	42	0	42	[26]
Sm‐doped 0.3BiFeO_3_‐0.7BaTiO_3_ (SPE)	152	80.0	5.0	75	10	65	[10]
BiFeO_3_‐BaTiO_3_‐SrTiO_3_ (RFE)	112	80.0	4.9	60	10	50	[24]
6 mol% Si‐doped HfO_2_ (AFE)	61	65.0	4.5	42	5	37	[27]
(Pb_0.97_La_0.02_)(Zr_0.66_Sn_0.23_Ti_0.11_)O_3_ (AFE)	46	84.0	4.0	66	10	56	[28]
Mn‐doped 0.4BiFeO_3_‐0.6SrTiO_3_ (RFE)	51	64.0	3.6	51	10	41	[29]
(Pb_0.97_La_0.02_)(Zr_0.98_Ti_0.02_)O_3_ (AFE→FE)	58	37.3	2.8	130	65	65	[30]
Bi_3.25_La_0.75_Ti_3_O_12_ (RFE)	45	78.4	2.0	60	8	52	[31]
(Pb_0.92_La_0.08_)(Zr_0.52_Ti_0.48_)O_3_ (FE)	22	77.0	1.6	70	30	40	[32]
Fe‐doped (Na_0.5_Bi_0.5_)TiO_3_ (RFE)	30	61.1	1.0	110	35	75	[33]
La‐doped PbZrO_3_ (AFE)	17	80.8	1.0	40	2	38	[34]
0.94(Bi_0.5_,Na_0.5_)TiO_3_‐0.06BaTiO_3_ (RFE)	86	81.0	1.7	131	7	124	This work

**Figure 3:**
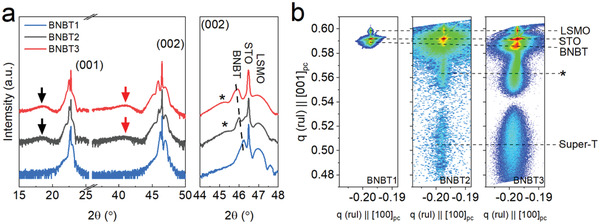
a) XRD *θ*‐2*θ* scan results and b) XRD‐RSM around (103) reflections of the BNBT thin films.

To understand the origin of the giant energy storage density under a moderate field, multi‐dimensional characterizations are carried out on the BNBT thin films. The out‐of‐plane X‐ray diffraction (XRD) *θ*–2*θ* scan patterns of BNBT thin films at room temperature are given in **Figure** **3**a, suggesting the preferred (*00l*) orientation of all thin films. The broad peaks on the diffraction patterns of BNBT2 and BNBT3 marked by black and red arrows may refer to the super‐T phase with significant expansion in the *c* axis.^[^ ^17^, ^22^ ^]^ BNBT1 is a coherently strained thin film without any additional peaks being visible on its XRD pattern. In the magnified patterns in the vicinity of the (002) diffraction peaks, the Laue fringes appearing to the right of the STO peak suggest the highly crystallized nature of the LSMO bottom electrode layer. The thin film (002) peaks shift to lower 2*θ* angles from BNBT1 to BNBT3, indicating the gradual increase in lattice parameter *c*. An extra peak marked with an asterisk on XRD patterns of BNBT2 and BNBT3 samples is also detected, implying the existence of transitional structures between normal and super‐T structures of BNBT. The XRD reciprocal space map (XRD‐RSM) scans around the (103) plane (Figure 3b) also suggest the presence of super‐T phases in BNBT2 and BNBT3 with a *c*/*a* ratio ranging from 1.11 to 1.25, greatly larger than that of the bulk ceramic counterparts possessing a perovskite structure with lattice parameters *a* = 0.3885 nm, *c* = 0.3903 nm, and *c*/*a* = 1.005,^[^ ^16^ ^]^ and any other available results for BNBT‐based thin films in literature. Another clue inferred from the RSM results lies in the higher concentration of the super‐T phase in BNBT3 than in BNBT2, given the stronger intensity of its super‐T diffraction spot in Figure 3b. The diffraction spots marked with asterisk arose from the transitional lattice between the normal BNBT lattice and the super‐T structures, consistent with the observation of XRD *θ*–2*θ* scan patterns. The average lattice parameters of BNBT thin films and super‐T structures calculated from the XRD results are summarized in Table S1 in the Supporting Information.

**Figure 4:**
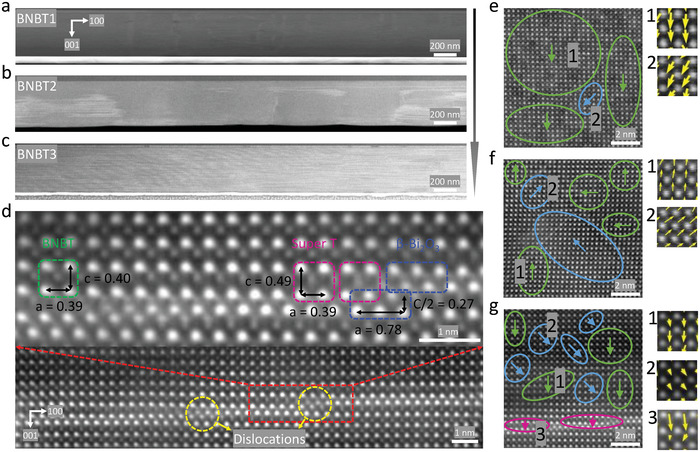
Low‐magnification HAADF‐STEM images of a) BNBT1, b) BNBT2, and c) BNBT3 show the different densities of Bi‐rich nanoclusters. d) Atomic resolution HAADF‐STEM image of BNBT3. The lattice parameters in the image are measured in nanometers. The domain orientation and distribution maps of e) BNBT1, f) BNBT2, and g) BNBT 3, where the areas of T, R, and super‐T phases are delineated by green, blue, and pink lines and arrows, respectively. The labeled numbers are the enlarged images to demonstrate the displacement of B‐site cations. It should be noted that the super‐T nanopolar clusters were intentionally not shown in the atomic resolution HAADF‐STEM image of BNBT2 for the purpose of better encapsulating their dominant polar structures.

High‐angle annular dark‐field (HAADF) scanning transmission electron microscopy (STEM) images were used to visualize the microstructures of the BNBT thin films on an atomic scale, as shown in **Figure** **4**. In HAADF‐STEM images, heavy elements exhibit bright contrast while light elements appear in dark contrast. Interestingly, various dimensions of chemical inhomogeneity are discovered in different BNBT films. Figure 4a–c demonstrates the microstructural evolution from BNBT1 to BNBT3 films. Highly localized Bi/Na enrichment areas (one to two atomic columns in dimension) are found in BNBT1 (Figure S5a, Supporting Information). In contrast, bright and gray stripes appear in BNBT2 grown at a lower temperature (Figure 4b). Further reducing growth oxygen pressure (*P*_O2_) (BNBT3 films) leads to a unique in‐plane superstructure consisting of alternating bright and gray stripes with a high density (Figure 4c and Figure S5b, Supporting Information), reflecting the formation of 2D elemental enrichment regions. Combining high‐resolution HAADF‐STEM imaging and atomic‐scale electron dispersive spectroscopy mapping (Figure S6, Supporting Information), the observed bright stripes are confirmed to be Bi‐rich atomic layers (half *β*‐Bi_2_O_3_ unit cell) belonging to a space group of *P‐4b2*.^[^ ^22^ ^]^

The increased density and dimension of bright and gray stripes from BNBT1 to BNBT3 films suggest that the deposition temperature and pressure play a critical role in chemical inhomogeneity in BNBT films. When both deposition temperature and *P*_O2_ are high, i.e., the case of BNBT1 thin film (750 °C and 200 mTorr), the adatoms will have enough time and kinetic energy to diffuse on the substrate and subsequently grow surfaces. Because of the intraplume scattering events during pulsed laser deposition,^[^ ^35^ ^]^ highly localized Bi/Na enrichment areas are produced without the formation of secondary phase. On the contrary, lower growth pressure and deposition temperature result in Bi‐rich layers, i.e., *β*‐Bi_2_O_3_ in BNBT2 and BNBT3 films. When lowering the oxygen partial pressure (100 mTorr) during the growth, atoms and clusters, including the excess Bi that is ablated from the target, will be less scattered by the O_2_ molecules in the LMBE chamber, thus favoring the deposition of excess Bi onto the substrate. Lower deposition temperature impedes the evaporation and diffusion along the freshly grown surface due to insufficient kinetic energy, resulting in the segregation of local Bi‐rich atomic layers, i.e., the *β*‐Bi_2_O_3_ phase. In addition, an atmosphere with deficient oxygen could facilitate the fast nucleation of *β*‐Bi_2_O_3_.^[^ ^17^ ^]^ As a result, the concentration of *β*‐Bi_2_O_3_ is gradually increased from BNBT1 to BNBT3 films.

Figure 4d shows the heteroepitaxial structure of BNBT/*β*‐Bi_2_O_3_ in BNBT3 films. The Bi‐rich atomic rows sustain over a few nanometers (max. 2–5 nm) instead of extending into a continuous layer at a long‐range distance, which is supported by the Z‐intensity profile results (Figure S7, Supporting Information) that clearly uncovers the transition from Bi atoms in *β*‐Bi_2_O_3_ to Ti atoms in BNBT, confirming the presence of the BNBT/*β*‐Bi_2_O_3_ interface in the *c*‐axis direction. The normal perovskite‐structured BNBT is found in the enlarged image while a stretched *c* lattice parameter of ≈0.49 nm in the BNBT unit cells is observed near the BNBT/*β*‐Bi_2_O_3_ interface, confirming the presence of super‐T phase. Given the fact that lattice parameter *a* of *β*‐Bi_2_O_3_ approximately doubles that of BNBT [*a_*β*_*_‐Bi2O3_ = 0.774 nm^[^ ^36^ ^]^ ≈ 2 *a*_BNBT_ (0.3885 nm)], a cube‐on‐cube heteroepitaxy is achieved with minimal in‐plane strain. However, owing to the large lattice mismatch in the *c* axis between *β*‐Bi_2_O_3_ (≈0.563 nm) and BNBT (≈0.3903 nm), the unit cells of both phases in the immediate proximity of BNBT/*β*‐Bi_2_O_3_ vertical interfaces suffer from a large interphase strain, i.e., *β*‐Bi_2_O_3_ unit cell experiences a compressive strain of ≈4% while BNBT unit cell is significantly stretched in the out‐of‐plane direction with an enormous tensile strain of ≈26%, resulting in the metastable super‐T phase of BNBT (*c*/*a* ratio of 1.25). Such substantial interphase strains relax quickly in the perimeter of BNBT/*β*‐Bi_2_O_3_ vertical interfaces through creating lattice defects, e.g., edge dislocations, as seen in Figure 4d, returning the *c* axis of BNBT to ≈0.40 nm, which is quite close to the bulk lattice. In this case, the super‐T phase of BNBT is mostly present in the form of quasi‐2D polar clusters of ≈5–8 nm in length, which is also reflected by the *c*/*a* ratio map in Figure S8 in the Supporting Information.

To gain a deeper insight into the relationships between nanostructures and outstanding energy storage performance, domains in the BNBT thin films with different phases and polarization directions were mapped out by colored arrows, as shown in Figure 4e–g. The observation of both tetragonal (T phase) and rhombohedral phases (R phase) in all the samples echoes the MPB composition. BNBT1 sample (Figure 4e) is composed of a matrix of T domains embedded with very few nano‐clustered R phase, giving rise to the responses of a typical ferroelectric, i.e., square *P*–*E* loop (Figure 2a) and distinct phase transition (Figures S1 and S3, Supporting Information). In contrast, BNBT2 displays intermediate‐sized nanodomains and nanopolar clusters compared with the other two samples, interpreting its nonergodic relaxor‐like properties, e.g., a slim *P*–*E* loop with substantial *P*_r_. BNBT3 demonstrates a mixture of nanopolar clusters of R, T, and super‐T phases, strongly underpinning the observed relaxor behaviors such as significantly reduced hysteresis and diffuse phase transition. Intriguingly, the presence of super‐T nanopolar clusters also enables a giant *P*_m_ that is even higher than the counterparts with large‐sized domains.^[^ ^37^ ^]^ Furthermore, the uniformly dispersed T, R, and super‐T nanoclusters in BNBT3, as illustrated in Figure 4g, suggest a weakly correlated polar state, which is beneficial for maintaining a low *P*_r_ and *E*_c_. Consequently, an unprecedentedly high (*P*_m_ – *P*_r_) is obtained in the studied BNBT3 thin films under a moderate electric field, greatly contributing to their giant energy storage density.

To further stress the critical role of super‐T nanopolar clusters in bolstering energy storage properties of glassy ferroelectric with MPB composition, phase field simulation was employed to simulate the polar evolutions of the studied BNBT thin films under the influence of an external electric field. The details of phase field simulations are given in the Experimental Section. A glassy ferroelectric having MPB composition with the coexistence of R + T nanostructures was set as the initial state in a perovskite single crystal, akin to BNBT1 sample. The impacts of the super‐T phase on the electrical properties were studied by introducing 3% and 15% of super‐T nanostructures into the initial state. The percentage of super‐T phase *x* remains consistent with that of the BNBT2 and BNBT3 thin films, in which the volume fraction of different phase structures was estimated based on transmission electron microscope (TEM) images (Table S2, Supporting Information). When the super‐T phase with a large *c/a* ratio is introduced, the local strain heterogeneity generated around super‐T nanoregions will decrease domain size with strong local random fields, allowing the strengthening of glass‐like features, e.g., the decrease of *P*_r_ and minimal hysteresis.

**Figure 5:**
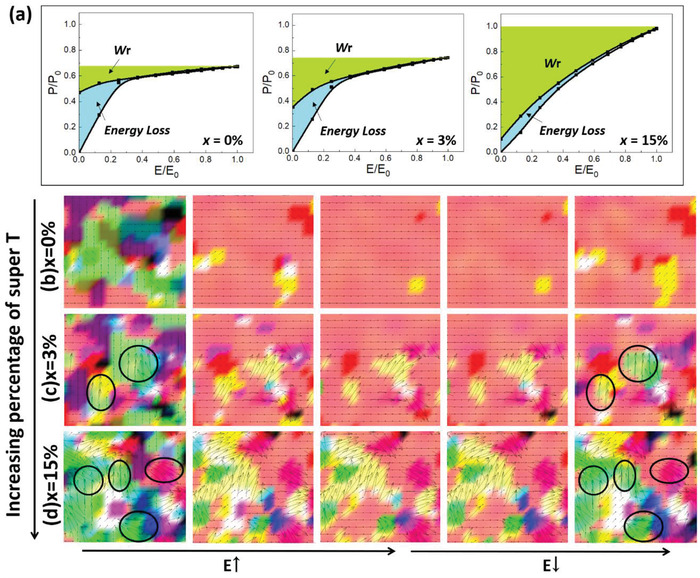
a) Phase‐field‐simulated initial cycle of *P*–*E* loops of a glassy ferroelectric with MPB composition and different percentage *x* of super‐T nanostructures (*x* = 0%, 3%, and 15%). The associated evolution of polar structures of b) *x* = 0, c) *x* = 3%, and d) *x* = 15% under a given field of *E*_0_ (> coercive field *E*_c_ of the three cases). *P*_0_ represents the maximum polarization of *x* = 15% under *E*_0_. The colors differentiate the ferroelectric domains with differently oriented polar directions. The super‐T domains are marked by black circles.

The calculated *P–E* loops and associated polar structure evolutions in response to the applied electric field (along [100] direction) of the three cases with different percentages of super‐T phases are shown in **Figure** **5**. In the absence of the super‐T phase (*x* = 0), the R + T nanodomains will transform into large ferroelectric domains aligned along the electric field direction (see Figure 5b). Due to the low local random field, the long‐range ordered ferroelectric domains will persist and not return to the initial state after the removal of the applied field, resulting in a canonical *P*–*E* loop of ferroelectrics as seen in BNBT1 films. When *x* = 3%, the strengthened local random field in glassy ferroelectrics will lead to a slimmer *P–E* loop compared to that of *x* = 0. For *x* = 15%, the *P–E* loop becomes the thinnest in the three cases due to the strongest local random field. The evolution of polar structure in *x* = 15% also clarifies that the initial state consisting of mixed R + T + super‐T nanostructures will be mostly recovered after the field is removed, indicative of a low *P*_r_. When *x* = 3%, on the other hand, only a small amount of initial domains is recovered after removing the field, leading to a *P*_r_ quantifying between the values of *x* = 0 and *x* = 15%. In line with the experimental results, the amplitude of *P*_m_ increases drastically with the increase in the volume fraction of the super‐T phase. Hence, the energy storage property is greatly benefited from the maximized (*P*_m_ – *P*_r_) and trivial *E*_c_, giving rise to the ultrahigh *W*_r_ in BNBT3 under a moderate electric field.

## 3 Conclusions

In this work, we have successfully developed a novel route to markedly improve the electrostatic energy storage density in dielectric thin films without the need for an excessive electric field. By introducing super tetragonal nanostructures into glassy ferroelectric with MPB composition, a giant energy storage density of ≈86 J cm^−3^ with a high energy efficiency of ≈81% was obtained under a moderate field of 1.7 MV cm^−1^ in a thin film of conventional ferroelectrics, i.e., 0.94(Bi, Na)TiO_3_‐0.06BaTiO_3_. The ultrahigh energy storage properties of the BNBT thin films demonstrated excellent thermal stability and fatigue endurance, confirming the viability of practical applications under a wide range of operating conditions. As suggested by the phase field simulation results and STEM observation, the presence of super‐T nanostructures around MPB composition generates an appreciable local strain heterogeneity, thus greatly suppressing the formation of long‐range ordered ferroelectric domains. Such a unique nanopolar structure leads to a very large maximum polarization *P*_m_ and a minimal hysteresis, strongly promoting a superb energy storage density while maintaining high energy efficiency. Our strategy based on super tetragonal nanopolar clusters could be readily generalized to many other perovskite‐structured relaxors ferroelectric thin films for optimizing not only energy storage, but also other functionalities including electrocaloric and pyroelectric properties.

## 4 Experimental Section

### Experimental

*Sample preparation*: The BNBT ceramic targets for thin film deposition were prepared by the conventional sintering process. 10 mol% excess of Bi_2_O_3_ was added to the target for compensating the loss of Bi during the sintering and subsequent laser ablation process. The trace amount of Mn (0.5 mol%) was also introduced into the BNBT target to minimize the leakage current in the resulted thin films. 400 nm thick BNBT thin films were grown on La_0.7_Sr_0.3_MnO_3_ (LSMO)‐electroded (001) SrTiO_3_ (STO) single‐crystal substrates (*a* = 0.3905 nm, cubic) by LMBE (PASCAL Combi, Japan). A KrF excimer laser (*λ* = 248 nm) with an energy density of 6.0 J cm^−2^ and frequency of 5 Hz was adopted for the deposition. Three BNBT thin films were fabricated with various deposition temperatures and oxygen partial pressures, i.e., 750 °C and 200 mTorr (BNBT1), 650 °C, and 200 mTorr (BNBT2), and 650 °C and 100 mTorr (BNBT3). Gold dot top electrodes with a 200 µm diameter were deposited through a shadow mask by a sputter coater (Leica EM ACE600) prior to the electrical measurements.

*Characterizations*: Temperature‐dependent ferroelectric hysteresis (*P–E*) loops were measured by the radiant ferroelectric workstation connected with a program‐controlled chamber (Linkam, HFS600E‐PB4). Temperature‐dependent dielectric properties were measured by an impedance analyzer (Agilent 4294A Precision). The room‐temperature XRD patterns and XRD‐RSM mapping were obtained by Smartlab (Rigaku, 9 kW rotating anode thin‐film XRD). Specimens for STEM observations were prepared by focused ion beam (FEI Helios G4 PFIB and Zeiss Auriga dual beam systems). Cross‐sectional lamellae were lifted out from the thin films and mounted to copper grids by platinum. Then the lamellae were thinned to the thickness allowing the electron transparency. HAADF‐STEM images were acquired using a HAADF detector in an aberration‐corrected FEI‐Themis Z operated at 300 kV. The images were obtained with a semiconvergence angle of 17.9 mrad and a collection angle range of 48–200 mrad. The STEM‐EDX map was acquired using the FEI Titan G2 ChemiSTEM operated at 200 kV. Local domain switching behaviors were studied by commercial PFM (Cypher S, Asylum Research, US) using Pt/Cr‐coated conductive probes (ElectriMulti 75G, BudgetSensors, Bulgaria).

### Details of Phase Field Simulations

The phase field simulations for the formation of R and T nanodomains are similar to our previous work.^[^ ^16^ ^]^ The formation and increase of super T nanodomains can be caused by the chemical disorder and related local field‐induced relaxor transition.^[^ ^38^ ^]^

The total free energy *F* of the model system included Landau free energy, *F*_Landau_ ($\int f_{\mathrm{Landau}}dV$), the gradient energy, *F*_grad_ ($\int f_{\mathrm{grad}}dV$), the elastic strain energy, *F*_elas_ ($\int f_{\mathrm{elas}}dV$), the electrostatic energy, *F*_ele_($\int f_{\mathrm{ele}}dV$), and extra energy caused by the local electric field from doping, *F*_LEF_($\int f_{\mathrm{LEF}}dV$)_,_ as described in Equation (1)

(1) $$F=\int f_{\mathrm{landau}}+f_{\mathrm{grad}}+f_{\mathrm{elas}}+f_{\mathrm{ele}}+f_{\mathrm{LFE}}dV$$

(2) flandau=fR+T+fSuperTflandau=A1R+T∑i3Pi2−A2R+T∑i3Pi22+A12R+T∑i,j,i≠j3Pi2Pj2flandau=+A13R+T∑i,j,i≠j3Pi4Pj2+A14R+TP12P22P32+A3R+T∑i3Pi23flandau=+A1SuperT∑i3Pi2−A2SuperT∑i3Pi22+A3SuperT∑i3Pi23

where *f*_R+T_ and *f*_SuperT_ stand for the Landau free energy for R and T nanodomain regions and super T nanodomain regions, respectively. The phase stability (C, T, R, Super T) could be described by the coefficient *A_i_* (*i* = 1–3), *A*_1_ *_j_* (*j* = 2–4), which depended on *c* (concentration) and T (temperature).^[^ ^39^, ^40^, ^41^ ^]^ The gradient energy density *f*_grad_ was written in terms of *P* (i.e., *P*_1_, *P*_2_, and *P*_3_) as follows$f_{\mathrm{grad}}=\frac{1}{2}\beta {\left(\sum_{i,j}^{3}P_{i,j}\right)}^{2}$ where *β* is the gradient energy coefficient. The elastic energy density *f*_elas_ was calculated by $f_{\mathrm{elas}}=\frac{1}{2}C_{ijkl}e_{ij}e_{kl}=\frac{1}{2}C_{ijkl}\left(\varepsilon_{ij}-\varepsilon_{ij}^{0}\right)\left(\varepsilon_{kl}-\varepsilon_{kl}^{0}\right)$, where *C_ijkl_* is the elastic constant, *e_ij_* is the elastic strain, *ε_ij_* is the total strain, and *ε_ij_* ^0^ is the spontaneous strain. The spontaneous strain was obtained from the polarization *P* by $\varepsilon_{ij}^{0}=Q_{ijkl}P_{k}P_{l}$, where *Q_ijkl_* is the electrostrictive coefficient. The electrostatic energy density *f*_ele_ was calculated by the equation $f_{\mathrm{ele}}=\sum_{i}^{3}-E_{i}P_{i}-\frac{1}{2}E_{i,\mathrm{depol}}\bar{P_{i}}$, where *E_i_* denotes the inhomogenous electric field due to the dipole–dipole interactions, *E_i_*_,depol_, the average depolarization field due to the surface charge, and $\bar{P_{i}}$ denotes the average polarization. The extra energy density caused by local electric field *f*_LEF_ was written by $f_{\mathrm{LFE}}=\sum_{i}^{3}-E_{i}^{\mathrm{LFE}}P_{i}$ . A set of random local electric fields with different orientations ($E_{1}^{\mathrm{LFE}},E_{2}^{\mathrm{LFE}},E_{3}^{\mathrm{LFE}}$ with Gaussian distribution $f\left(E_{i}^{\mathrm{LFE}}\right)=0.2e^{\left(-0.008{\left(E_{i}^{\mathrm{LFE}}-11\right)}^{2}\right)}$) was used to describe the local field in the simulations.^[^ ^38^ ^]^ The temporal dependence of the spontaneous polarization could be obtained by solving the time‐dependent Ginzburg–Landau function given by

(3) $$\frac{dP_{i}\left(r,t\right)}{dt}=-M\frac{\delta F}{\delta P_{i}\left(r,t\right)}+\varsigma ,i=1,2,3$$

where *M* is the mobility coefficient and *t* is the time, $\varsigma =\sqrt{2k_{B}TM/\left(l_{0}^{3}\Delta t\right)\rho }$ describes the thermal fluctuation and is adjusted by the random number *ρ* in simulations.^[^ ^42^ ^]^

The parameters used in the calculations were chosen according to refs. [16, 43, 44] and the landau coefficient *A*_1_^R+T^ = 4.124 × 10^5^, *A*_2_^R+T^ = 5 × 10^8^, *A*_12_^R+T^ = 8 × 10^8^, *A*_3_^R+T^ = 1.294 × 10^9^, *A*_13_^R+T^ = 1.5 × 10^9^, *A*_14_^R+T^ = 7.5 × 10^8^ and *A*_1_^SuperT^ = 3.716 × 10^6^, *A*_2_^SuperT^ = 4.05 × 10^8^, *A*_12_^SuperT^ = 6.48 × 10^8^, *A*_3_^SuperT^ = 0.91 × 10^8^ (all in SI units) were used in the simulations. Other related coefficients used in the simulations are given as below: elastic constants *C*_11_ = 1.78 × 10^11^ N m^−2^, *C*_12_ = 9.64 × 10^10^ N m^−2^, and *C*_44_ = 1.22 × 10^11^ N m^−2^; electrostrictive coefficients *Q*_11_ = 0.10 C^−1^ m^2^, *Q*_12_ = −0.034 C^−1^ m^2^, and *Q*_44_ = 0.029 C^−1^ m^2^; spontaneous polarization *P*_0_ = 50 µC cm^−2^ for normal T and R phase, *P*_0_ = 150 µC cm^−2^ for super T phase; external electric field *E*_0_ = 60 kV cm^−1^; the domain wall energy for 90^o^ domain walls ≈0.01 J m^−2^ and the domain wall mobility *M* = 4 × 10^4^ C^2^ ms^−1^.^[^ ^45^ ^]^ The dimensionless parameters used in the simulations were chosen according to the energy scaling factor *∆f*_scale_ = 10^8^ J m^−3^.

## Conflict of Interest

The authors declare no conflict of interest.

## Supporting information

Supporting Information

## Data Availability

The data that support the findings of this study are available from the corresponding author upon reasonable request.
