# Supplementary material for: Ultrahigh Energy Storage Density in Glassy Ferroelectric Thin Films under Low Electric Field
Source: Adv Sci (Weinh). 2022 Sep 18;9(31):2203926. doi: 10.1002/advs.202203926 (PMC9631080; doi:10.1002/advs.202203926)
Supplement: Supplementary file 1 — Supporting Information [file ADVS-9-2203926-s001.pdf]

WILEY-VCH

Supporting Information

**Ultrahigh energy storage density in glassy ferroelectric thin films under low electric field**

*Yunlong Sun*[†]*, Le Zhang*[†,] **, Qianwei Huang, Zibin Chen*, Dong Wang, Mohammad Moein Seyfouri, Shery L. Y. Chang, Yu Wang, Qi Zhang, Xiaozhao Liao, Sean Li*, Shujun Zhang and Danyang Wang**

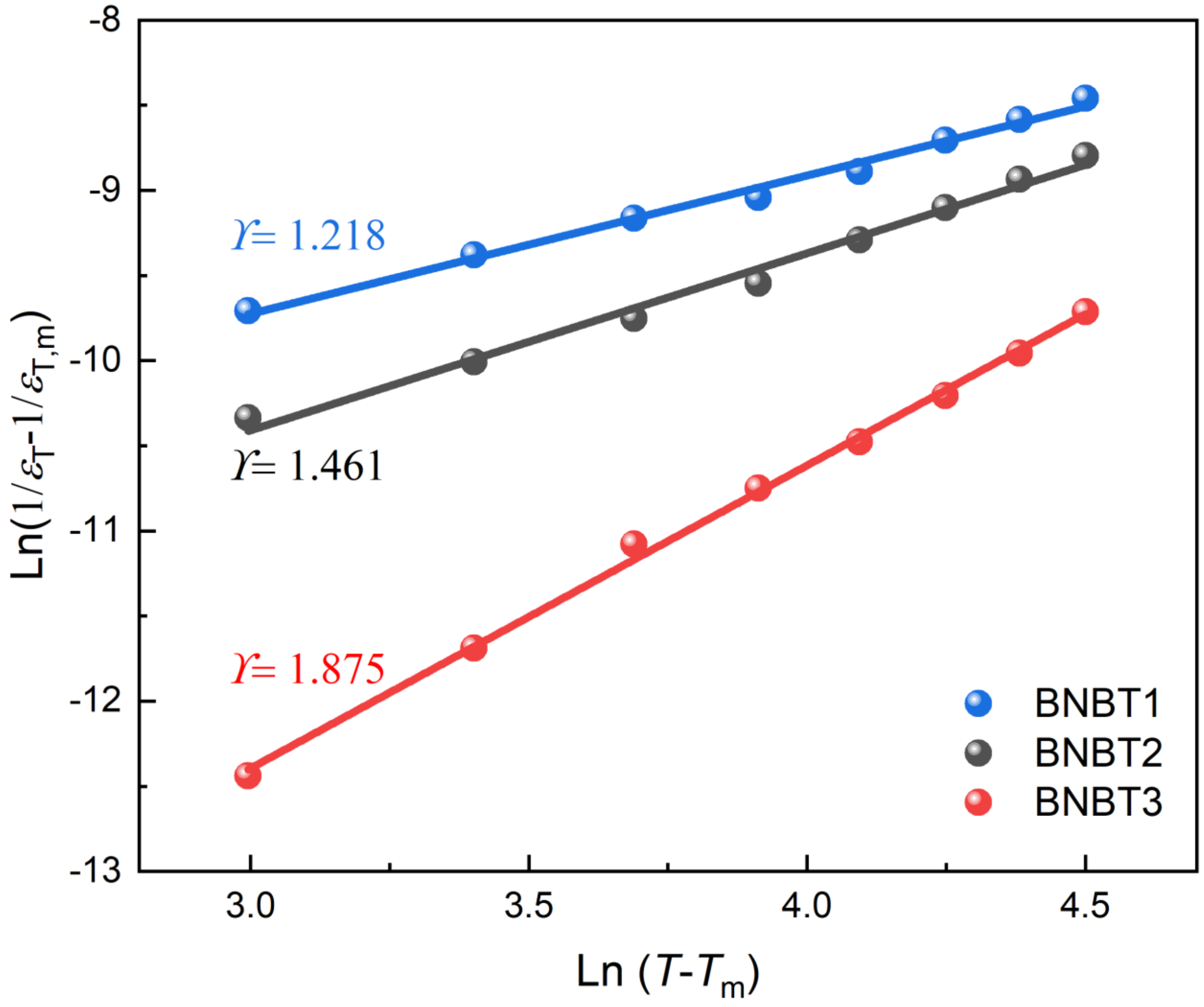


**Figure S1**. The relaxor diffuseness factor γ of BNBT thin films, which is derived from the modified Curie-Weiss law based on the temperature-dependent dielectric permittivity.

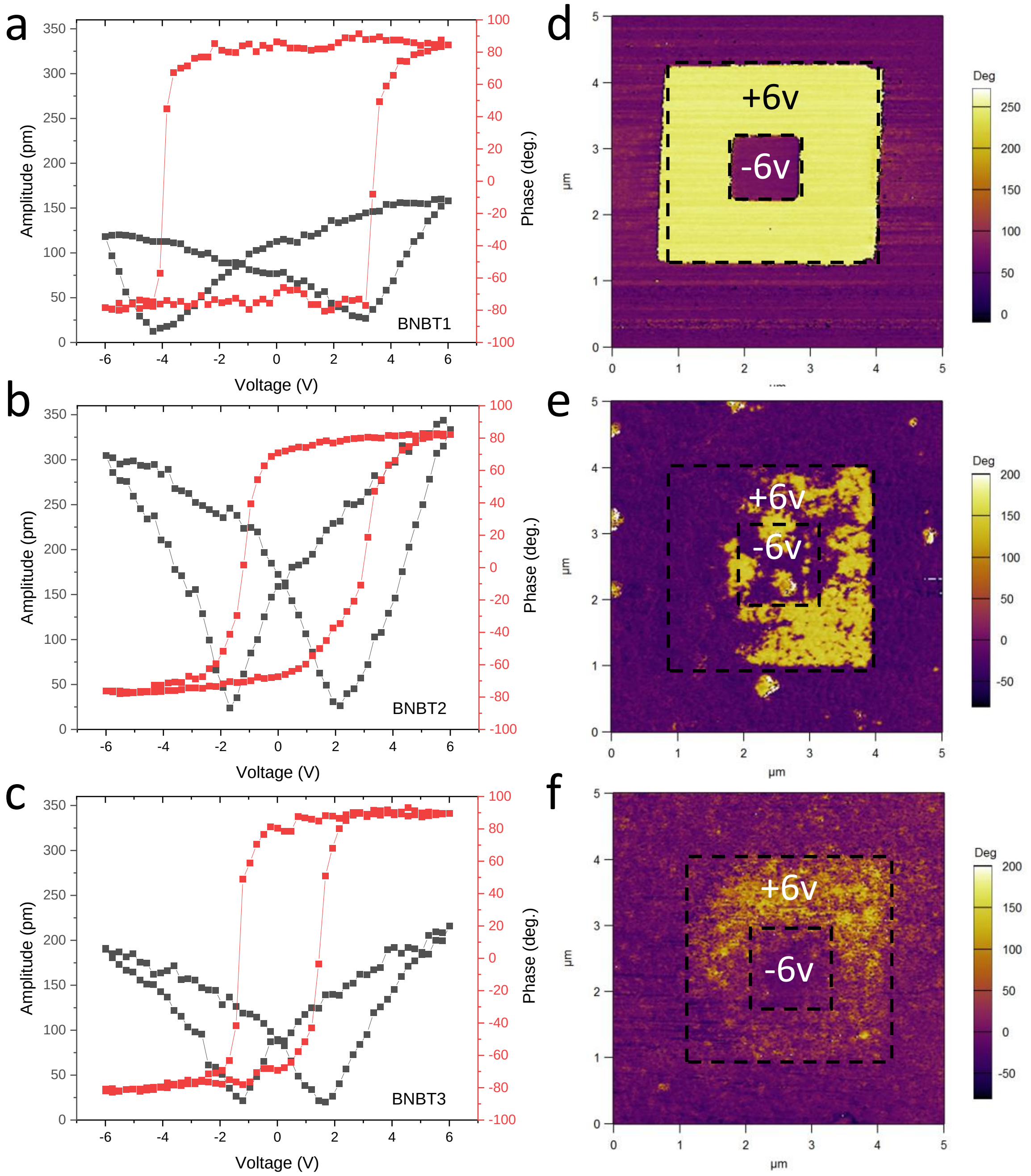


**Figure S2.** Local switching spectroscopy PFM amplitude-voltage butterfly loops and phase-voltage hysteresis loops for a) BNBT1, b) BNBT2 and c) BNBT3. Out-of-plane (OOP) PFM box-in-box phase images obtained immediately after DC poling for d) BNBT1, e) BNBT2 and f) BNBT3 thin films. Dual AC resonance tracking (DART) PFM (Cypher, Asylum Research) was used for these measurements. Yellow and purple contrasts indicate downward and upward polarization, respectively.

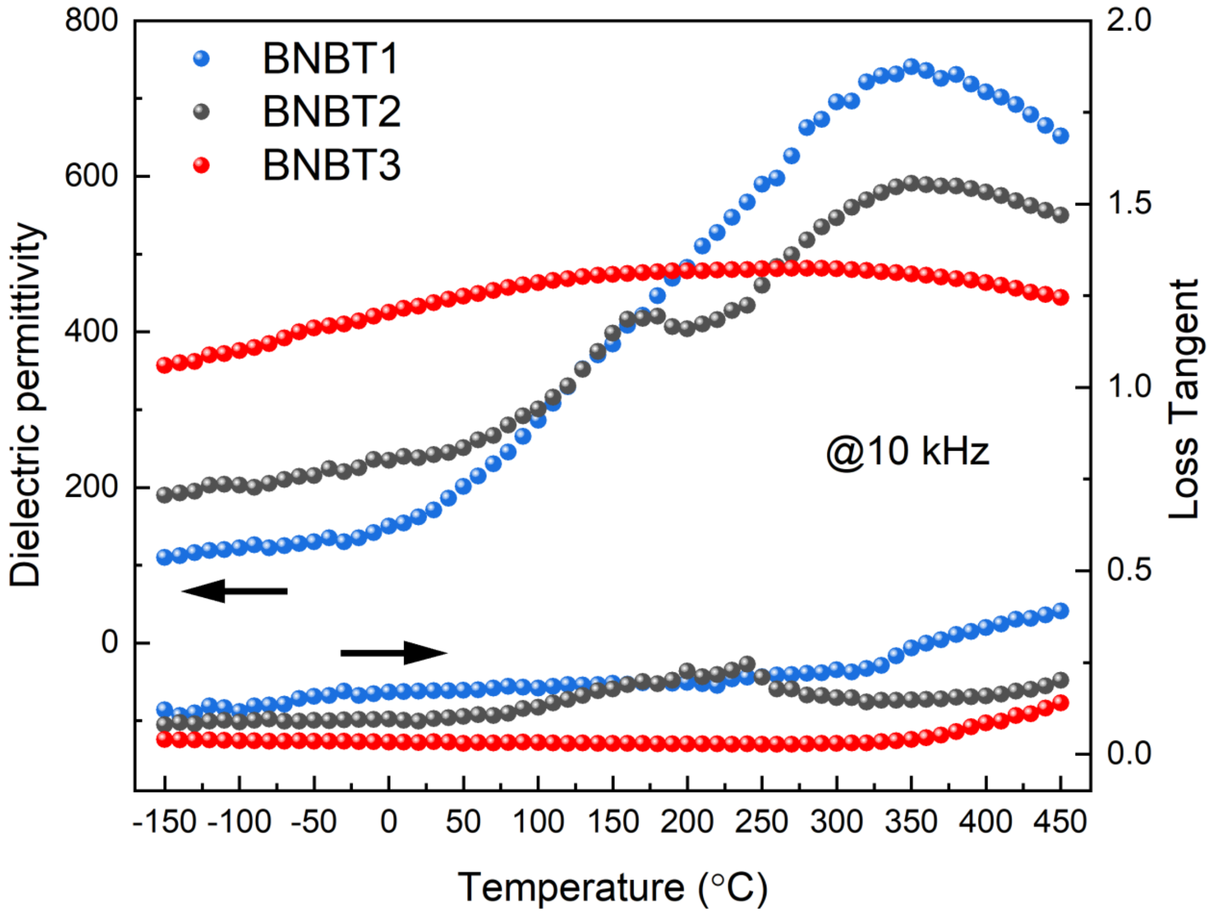


**Figure S3**. The temperature-dependent dielectric properties of BNBT thin films at 10 kHz from -150 to 450 $^{o}$C.

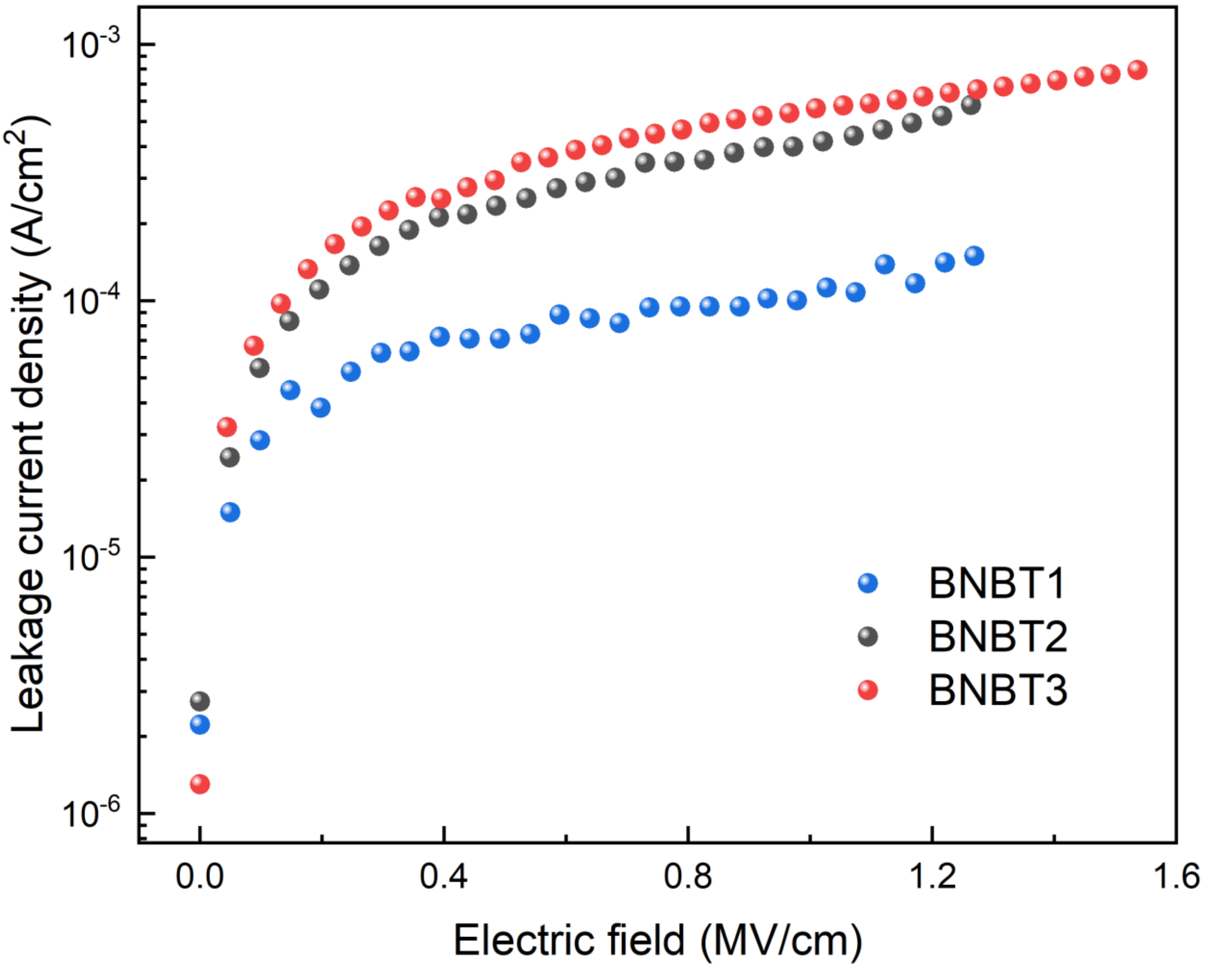


**Figure S4**. The leakage current density of BNBT films at room temperature.

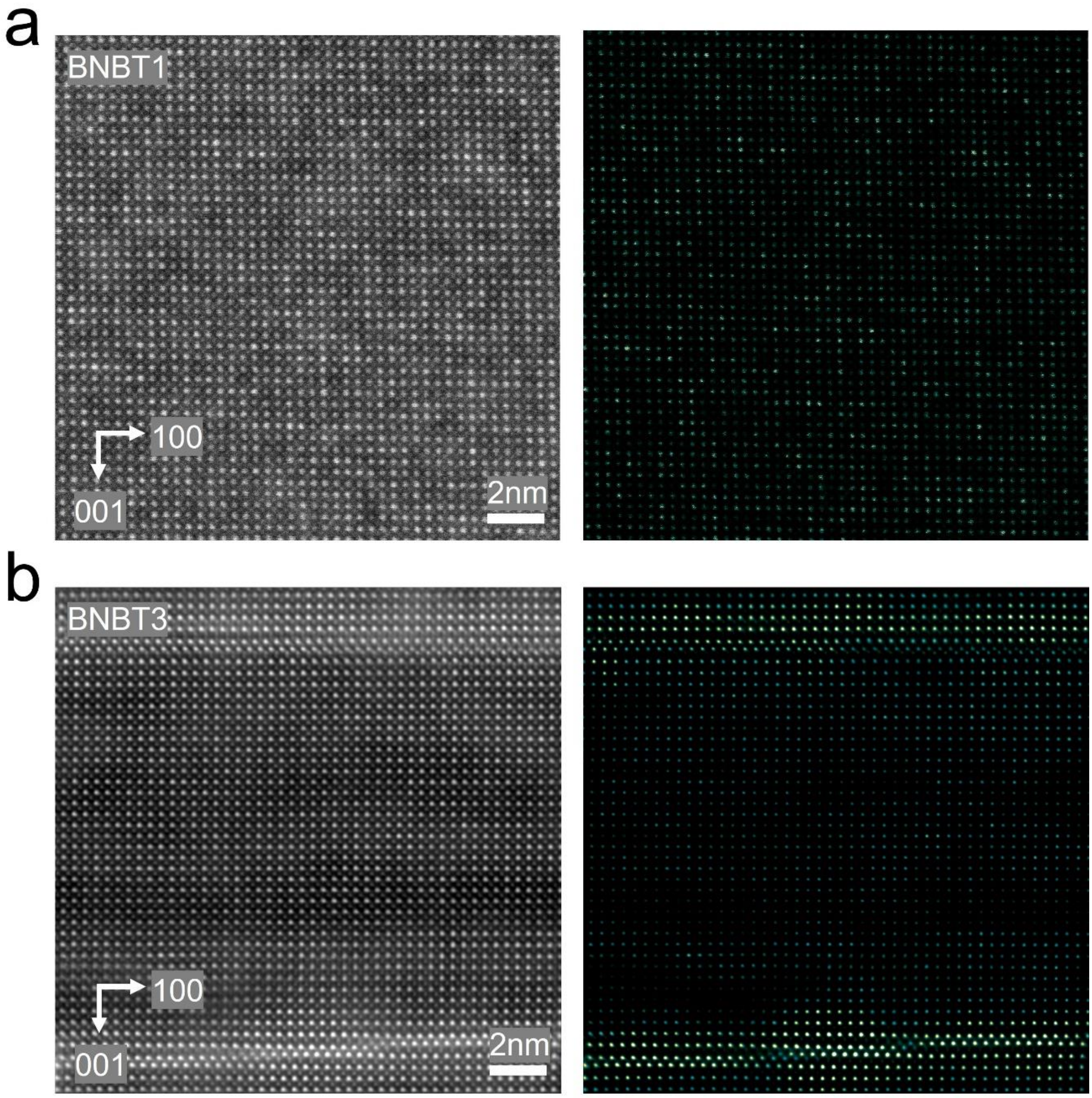


**Figure S5**. The atoms enrichment analysis of a) BNBT1 and b) BNBT3, where the yellow dots represent Bi enriched lattice sites.

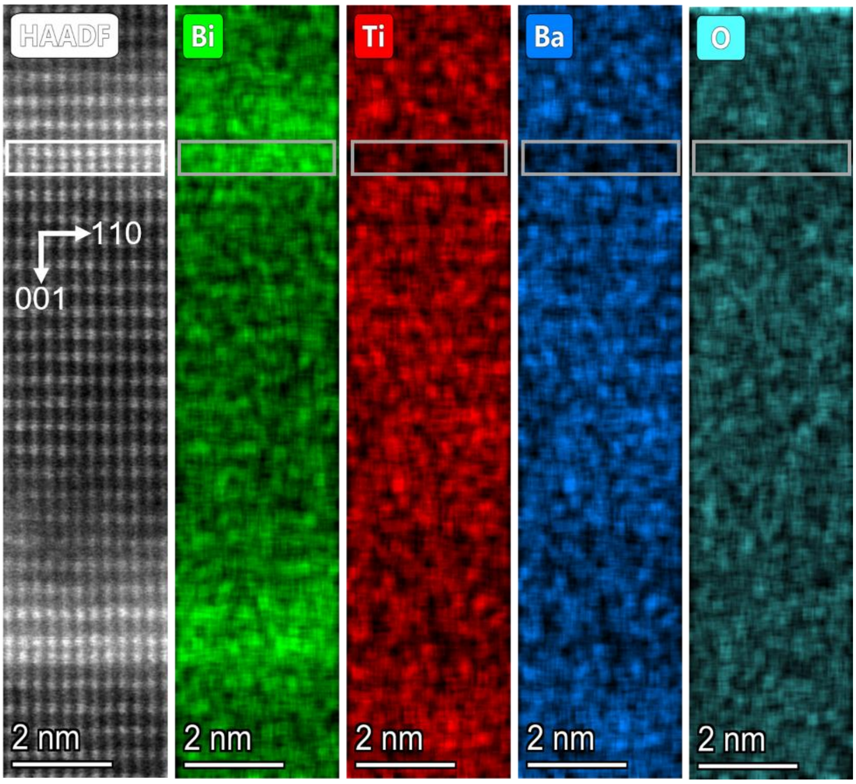


**Figure S6.** STEM-EDS elemental mapping of BNBT3 thin film. Ti and Ba are barely observed in the framed region, in which Bi is enriched, confirming the presence of the $BiO_x$ phase.

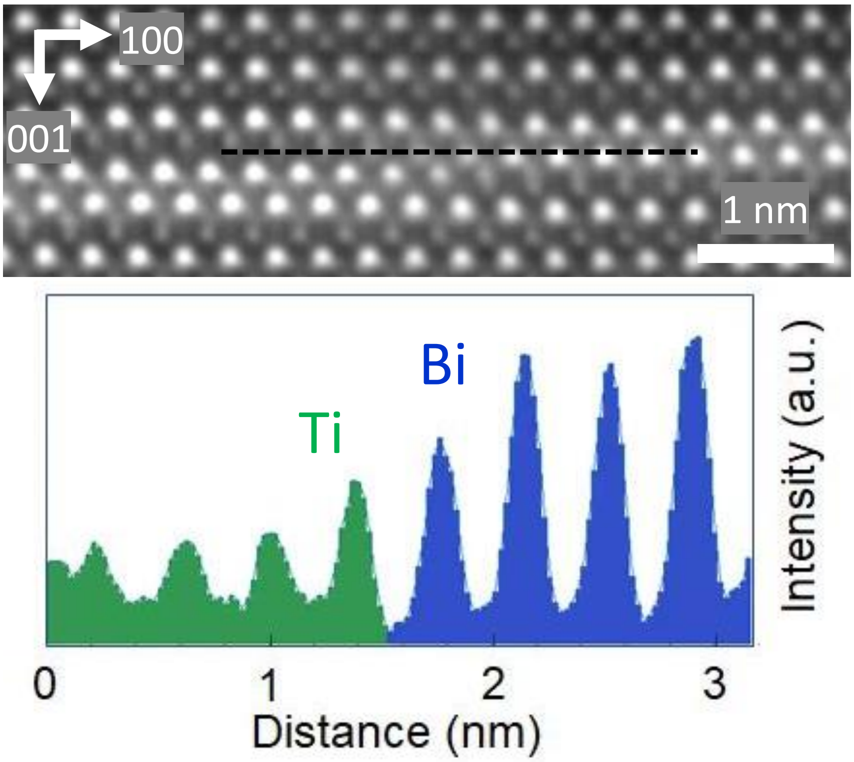


**Figure S7**. The HAADF atomic column intensity profile near the BNBT/*β*-$Bi_2O_3$ interface in the *c*-axis direction, showing the transition from Ti to Bi.

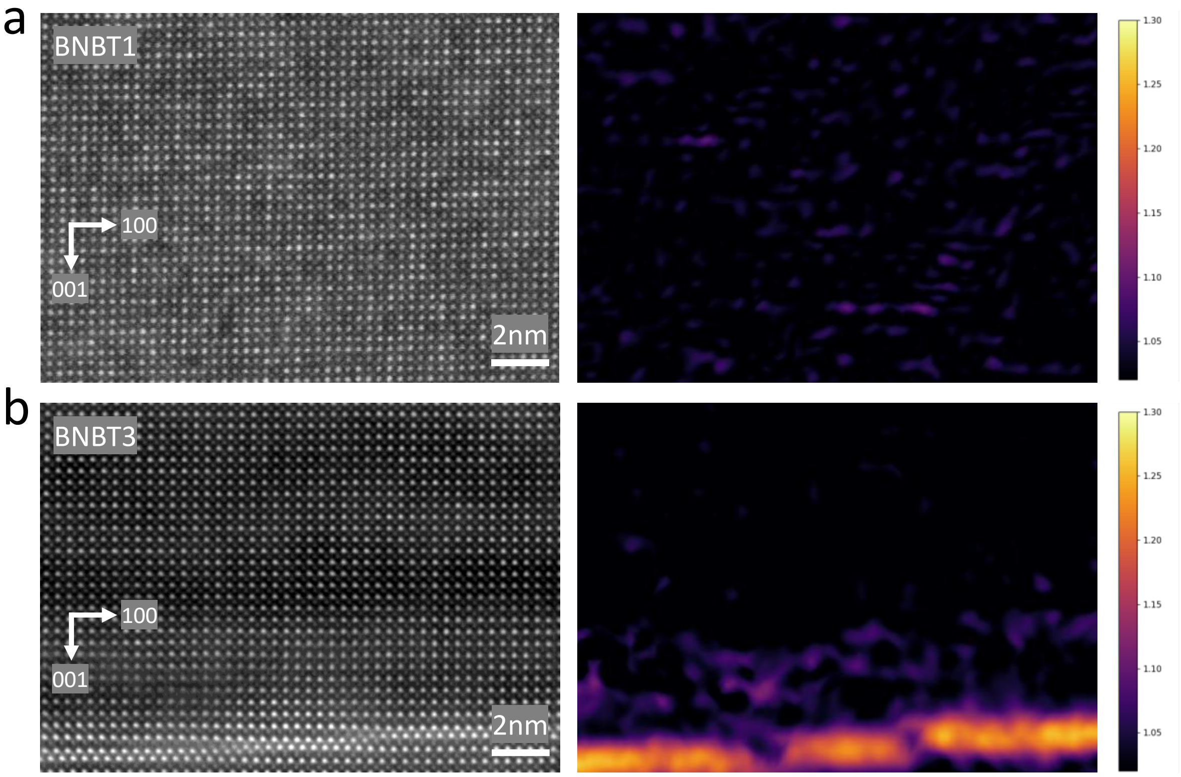


**Figure S8**. The *c*/*a* ratio map of a) BNBT1 and b) BNBT3.

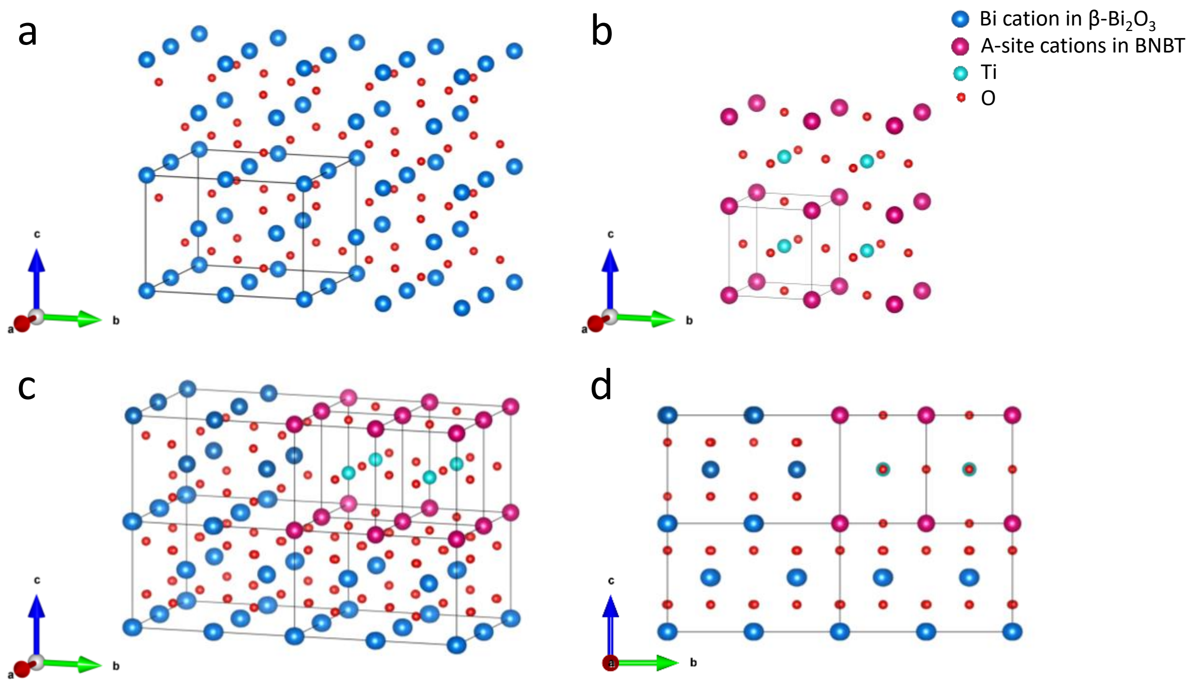


**Figure S9.** The schematic lattice structure for super-T and *β*-$Bi_2O_3$, where a) the lattice structure of *β*-$Bi_2O_3$, b) the lattice structure of BNBT, c) and d) the interface schematic of super-T and *β*-$Bi_2O_3$.

Determination of the volume fraction of super-teragonal phase in phase-field simulation

The volume fraction of the super-tetragonal phase used in the phase-field simulation was determined using the STEM images based on the line intercept method, which is a common approach to evaluate the dislocation density in alloys.[1, 2] The volume fraction of the super-tetragonal (super T) phase can be calculated through the following equation:

$$V_{super-T} = \frac{L_{super-T} \times T_{super-T}}{L_{lines} \times T_{sample}} = \frac{n \times l_{super-T} \times T_{super-T}}{L_{lines} \times T_{sample}}$$

where $V_{super-T}$ is the volume fraction of the super-T structure. $L_{super-T}$ and $L_{lines}$ are the total length of the super-T structure and the total length of the grid lines, respectively. $T_{super-T}$ and $T_{sample}$ are the in-plane thickness of the super-T structure and the thickness of the sample, respectively. $n$ is the number of intersections that super-T structures make with the grid lines, and $l_{super-T}$ is the unit length of the super-T structures per single intersection. Taking the BNBT3 sample as an example, multiple lines (15x in our analysis here) oriented along the [001] direction are applied and evenly distributed on the HAADF-STEM image, as shown in **Figure S10**a. According to the relative atomic mass, an intensity profile could be obtained from each line, where the peak represents one intersection which super-T structure makes with the grid line. By calculating the total number of the peaks, we can obtain the total number of intersections $n$. (Figure S10b). From the high-resolution STEM image (Figure 4d in the manuscript), we know that two atomic layers (the length of each atomic layer is 0.49 nm) of super-T BNBT will be formed near each intersection. Therefore, the unit length of the super-T structures per single intersection $l_{super-T}$ is known to be 0.98 nm. The total length of the grid lines is known and measurable. Because the super-T structure is a two-dimensional structure and the sample is very thin, the super-T structure is assumed to extend through the sample thickness, making $T_{super-T}=T_{sample}$. Based on the aforementioned approach, the volume fraction of the super-T phase in BNBT2 and BNBT3 thin films is estimated to be ~3% and ~15%, respectively.

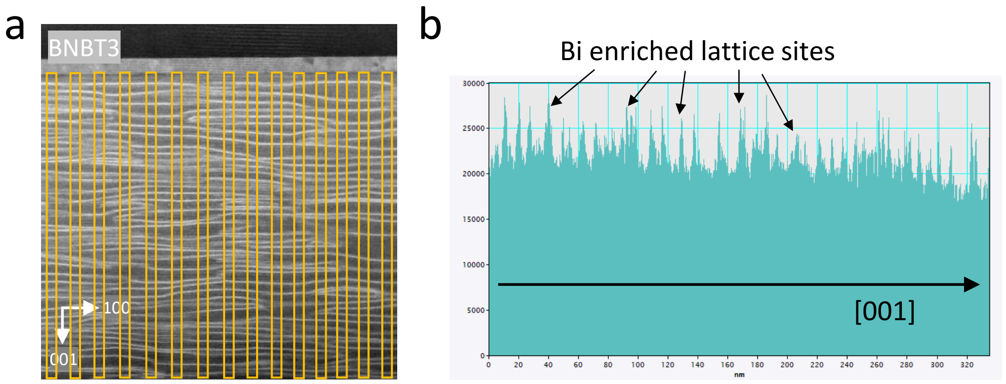


**Figure S10.** a) Schematic diagram of determining the volume fraction of super-T phase in BNBT thin films by line intercept method based on HAADF-STEM image. b) A typical intensity profile along the lines in (a).

**Table S1.** The summary of lattice parameters of BNBT thin films from the XRD results.

| Samples | Phase structure | *a* (nm) | *c* (nm) | *c*/*a* |
|---|---|---|---|---|
| BNBT1 | BNBT | 0.3902 | 0.3924 | 1.01 |
| BNBT2 | BNBT | 0.3902 | 0.3940 | 1.01 |
| | Super-T | 0.3852 – 0.3930 | 0.4328 – 0.4862 | 1.11 – 1.25 |
| BNBT3 | BNBT | ~0.3891 | 0.3949 | ~1.02 |
| | Super-T | 0.3787 – 0.3949 | 0.4328 – 0.4862 | 1.11 – 1.25 |

*When calculating the *c*/*a* super-T phases, *a* value is fixed at 0.3905 nm

**Table S2.** The percentage of regular R, T, and super-T phases in three BNBT samples estimated from TEM images.

| Samples | BNBT1 | | BNBT2 | | | BNBT3 | | |
|---|---|---|---|---|---|---|---|---|
| Phase structure | T | R | T | R | Super T | T | R | Super T |
| Volume percentage(%) | 90 | 10 | 82 | 15 | 3 | 55 | 30 | 15 |

Reference

[1] Y. Meng, X. Ju, X. Yang, *Mater. Charact.* **2021,** *175*, 111065.

[2] R. Ham, *Philos. Mag.* **1961,** *6* (69), 1183-1184.
